# Allelic prevalence and geographic distribution of cerebrotendinous xanthomatosis

**DOI:** 10.1186/s13023-022-02578-1

**Published:** 2023-01-17

**Authors:** Tiziano Pramparo, Robert D. Steiner, Steve Rodems, Celia Jenkinson

**Affiliations:** 1 Travere Therapeutics, Inc., 3611 Valley Centre Dr Suite 300, San Diego, CA 92130 USA; 2 Department of Pediatrics, University of Wisconsin School of Medicine and Public Health, 600 Highland Ave, Madison, WI 53792 USA

**Keywords:** Allele frequency, Rare metabolic disease, Cerebrotendinous xanthomatosis (CTX), Genomic variation, Pathogenic variants

## Abstract

**Background:**

Cerebrotendinous xanthomatosis (CTX) is a rare recessive genetic disease characterized by disruption of bile acid synthesis due to inactivation of the *CYP27A1* gene. Treatment is available in the form of bile acid replacement. CTX is likely underdiagnosed, and prevalence estimates based on case diagnosis are probably inaccurate. Large population-based genomic databases are a valuable resource to estimate prevalence of rare recessive diseases as an orthogonal unbiased approach building upon traditional epidemiological studies.

**Methods:**

We leveraged the Hardy–Weinberg principle and allele frequencies from gnomAD to calculate CTX prevalence. ClinVar and HGMD were used to identify high-confidence pathogenic missense variants and to calculate a disease-specific cutoff. Variant pathogenicity was also assessed by the VarSome implementation of the ACMG/AMP algorithm and the REVEL in silico predictor.

**Results:**

CTX prevalence estimates were highest in Asians (1:44,407–93,084) and lowest in the Finnish population (1:3,388,767). Intermediate estimates were found in Europeans, Americans, and Africans/African Americans (1:70,795–233,597). The REVEL-predicted pathogenic variants accounted for a greater increase in prevalence estimates for Europeans, Americans, and Africans/African Americans compared with Asians. We identified the most frequent alleles designated pathogenic in ClinVar (p.Gly472Ala, p.Arg395Cys), labeled pathogenic based on sequence consequence (p.Met1?), and predicted to be pathogenic by REVEL (p.Met383Lys, p.Arg448His) across populations. Also, we provide a prospective geographic map of estimated disease distribution based on *CYP27A1* variation queries performed by healthcare providers from selected specialties.

**Conclusions:**

Prevalence estimates calculated herein support and expand upon existing evidence indicating underdiagnosis of CTX, suggesting that improved detection strategies are needed. Increased awareness of CTX is important for early diagnosis, which is essential for patients as early treatment significantly slows or prevents disease progression.

**Supplementary Information:**

The online version contains supplementary material available at 10.1186/s13023-022-02578-1.

## Introduction

Cerebrotendinous xanthomatosis (CTX, OMIM 213700) is a rare autosomal recessive condition characterized by disruption of bile acid synthesis due to inactivation of the *CYP27A1* gene. Biallelic pathogenic variants are responsible for loss of enzymatic sterol-27-hydroxylase activity leading to reduced production of chenodeoxycholic acid (CDCA) and cholic acid, and accumulation of cholestanol and bile alcohols [1, 2]. CTX is highly heterogeneous in clinical presentation and signs and symptoms can overlap with other conditions (eg, sitosterolemia, familial hypercholesterolemia). The first signs and symptoms of the classical presentation are typically non-neurological and may include diarrhea during infancy (sometimes with failure to thrive), juvenile-onset-cataracts, and tendon xanthomas [3, 4]. Neurological dysfunction may be present in childhood with intellectual disability and/or autism spectrum disorder and frequently develops in adulthood with epilepsy, pyramidal and extrapyramidal signs, cerebellar syndrome, peripheral neuropathy and intellectual disability/cognitive decline and progressive neurodegeneration [4–10]. Because of the pleiotropic phenotypes, accurate diagnosis may be delayed 10–20 years [11, 12] and is often achieved only after irreversible neurological involvement has ensued. Treatment with bile acid replacement in the form of CDCA has been successfully used for decades [2, 13] and, alternatively, cholic acid treatment has been reported in a modest number of cases.

Clinical studies have provided powerful evidence showing that early recognition and treatment are critical to prevent or ameliorate disease progression and to avoid devastating neurological complications. Improved outcome was reported in two independent studies for patients with treatment initiated before age 24–25 years as compared to those patients who started later [14, 15]. These results further emphasize the need to improve early recognition strategies [16], stimulate newborn screening programs [17–19], and leverage large population genetic data to update and more accurately estimate disease prevalence [20].

A relatively recent approach of estimating disease prevalence from the causative or “predicted” causative alleles (herein labelled allelic prevalence) may contribute to increased disease awareness and improved surveillance and, ultimately, may stimulate earlier diagnosis [21–25]. This approach has become possible due to the availability of large population databases, such as the Genome Aggregation Database (gnomAD) [26]. Essentially, this genetic approach is unbiased and orthogonal to clinical studies that instead focus on the recognition, recruitment, and count of the number of confirmed affected individuals. This latter approach may suffer from several sources of bias, likely leading to inaccurate estimates and severe underdiagnosis of the disease. A clear limitation to disease recognition underlying failure to diagnose is the presence of “milder” phenotypes, which includes those CTX patients without neurological involvement [27]. Despite identification of the same underlying genetic variants observed in severe cases, such individuals may be overlooked and/or misdiagnosed with other, often more common, diseases. The identification and reporting of such patient subpopulations further support the notion that genotype–phenotype correlations have not been established in CTX [3].

A previously published first estimate of the genetic prevalence of CTX utilized data from the Exome Aggregation Consortium (ExAC) database, which included ~ 60,000 unrelated individuals [20]. Here we provide an update of genetic prevalence utilizing the much larger gnomAD [26] and taking advantage of additional resources, including the Human Gene Mutation Database (HGMD®) and ClinVar database as well as the VarSome variant classifier. Our approach to CTX prevalence estimate utilizes the Hardy–Weinberg principle and observed variant allele frequencies implementing a variety of variant inclusion/exclusion criteria and automated and manual genetic variant curation steps in an attempt to provide the most conservative and accurate result. In concordance with previous estimates, we report heterogeneity in prevalence across ancestries and identify the most frequent alleles driving these estimates. Finally, we present a map of worldwide geographic estimate disease distribution using a novel approach based on *CYP27A1* variant queries performed by clinicians across six continents. The map and CTX prevalence estimate results indicate that the number of individuals with this treatable disorder worldwide should greatly exceed the few hundred currently reported [12, 28].

## Methods

### Identification of high-confidence CYP27A1 pathogenic missense variants for cutoff calculation

We defined “high-confidence” (HC) pathogenic, missense variants classified as such by both the ClinVar database (https://www.ncbi.nlm.nih.gov/clinvar/) and the HGMD (http://www.hgmd.cf.ac.uk/ac/index.php) as previously adopted [29]. Only HGMD missense variants with a disease-causing mutation (DM) label that were also present and designated (likely) pathogenic in ClinVar were included in the cutoff calculation (see Additional file 1). We reclassified these HC pathogenic variants with the VarSome American College of Medical Genetics and Genomics (ACMG)/Association for Molecular Pathology (AMP)–based algorithm (https://varsome.com/about/resources/acmg-implementation/) and considered only those classified (likely) pathogenic (see Additional file 1). Among the HC pathogenic variants, we included the ClinVar “likely pathogenic” (LP) based on the following considerations: (1) according to the ACMG/AMP guidelines, LP correlates to a probability of 90% that a variant will be disease-causing [30]; (2) DM variants reported in HGMD also included LP according to the VarSome classification and were consistent with ClinVar labels; (3) the difference in interpretation confidence between pathogenic and LP was deemed medically insignificant [31]; and (4) retaining LP variants in our cutoff calculation did not support the hypothesis of lower confidence in pathogenic variant effects for LP. LP variants for the purposes of this study are included as pathogenic.

### Identification of CYP27A1 pathogenic variants in gnomAD for prevalence calculation

The gnomAD v2.1.1 (https://gnomad.broadinstitute.org/) was accessed on October 12, 2021, to gather *CYP27A1* variant information based on the canonical transcript ENST00000258415.4 and reference sequences NM_000784.4 and NP_000775.1. ClinVar clinical significance annotation in gnomAD was based on the October 2, 2021, release. We selected *CYP27A1* variants with a pathogenic ClinVar clinical significance label for all available sequence consequences. We manually verified that these variants were assigned pathogenic in the HGMD (DM) and the ClinVar database. In ClinVar, we considered reliable pathogenic submissions, including single submitters, limited to those from clinical genetic/diagnostic laboratories, since variant classification/interpretation was likely performed following ACMG/AMP guidelines [32]. All remaining frameshift, nonsense, start_lost, stop_gained, and canonical splice variants (2 bases ± splice acceptor/donor site) in gnomAD were considered pathogenic based on their high-impact consequence on the coding sequence (see Table 1), as calculated by VEP (https://m.ensembl.org/info/genome/variation/prediction/predicted_data.html) and classified in previous studies [25, 33]. To increase stringency, we manually reclassified and verified each pathogenic variant with the VarSome algorithm (see Additional file 1). Lastly, we filtered out variants with a gnomAD label of “lc_lof” and variants lacking multiple ClinVar entries from clinical genetic/diagnostic laboratories supporting pathogenicity.

**Table 1:** *CYP27A1* gnomAD variants

Variant type	All N (%)	ClinV-P N (%)	SeqCon-P N (%)	Revel-P N (%)	Model N (%)
Missense	344 (39.8)	13 (3.8)	–	28 (8.1)	41 (11.9)
Intron	232 (26.9)	–	–	–	–
Synonymous	139 (16.1)	–	–	–	–
Splice_region	35 (4.1)	–	–	–	–
Frameshift	31 (3.6)	12 (38.7)	17 (54.8)	–	29 (93.5)
Stop_gained	23 (2.7)	11 (47.8)	10 (43.5)	–	21 (91.3)
5_prime_UTR	20 (2.3)	–	–	–	–
3_prime_UTR	18 (2.1)	–	–	–	–
Splice_donor	8 (0.9)	5 (62.5)	3 (37.5)	–	8 (100)
Inframe_deletion	4 (0.5)	–	–	–	0 (0. 0)
Splice_acceptor	4 (0.5)	3 (75.0)	1 (25.0)	–	4 (100)
Start_lost	3 (0.3)	–	3 (100)	–	3 (100)
Inframe_insertion	2 (0.2)	–	–	–	–
Stop_retained	1 (0.1)	–	–	–	–
Total	864 (100)	44 (5.1)	34 (3.9)	28 (3.2)	106 (12.3)

Variant count breakdown by type: all gnomAD variants (All), designated pathogenic based on ClinVar clinical significance label (ClinV-P); considered pathogenic based on sequence consequence (SeqCon-P); predicted pathogenic based on REVEL analysis (Revel-P), and final list of variants selected for the prevalence calculation (Model)

### Identification of CYP27A1 predicted pathogenic variants in gnomAD for prevalence calculation

All remaining missense variants in gnomAD (N = 321) underwent bioinformatic analysis to predict the likelihood of pathogenicity. We used the REVEL ensemble in silico predictor, which predicts the pathogenic variant effects of missense based on a combination of scores from 13 individual tools and is considered more reliable in assessing pathogenicity [34] than the historically popular SIFT and Polyphen-2 tools [30]. REVEL is the reference algorithm for the ACMG/AMP variant interpretation as part of the standard operating procedure in the SVI-approved expert panel specifications (https://clinicalgenome.org/working-groups/sequence-variant-interpretation/).

We established a disease-specific cutoff using the mean REVEL score value calculated from the list of HC pathogenic missense variants defined above (see Additional file 1). The REVEL mean value was calculated after removing outliers using the interquartile range formula, as previously adopted [20]. The calculated score (0.834) was then used as cutoff to filter the remaining 321 missense variants. Only gnomAD missense variants with scores equal to or greater than the calculated REVEL cutoff were predicted pathogenic and retained in the complete model for the prevalence calculation. Lastly, to avoid underestimation of the prevalence calculation, we manually investigated the excluded missense variants and recovered those with ClinVar submissions supporting evidence for pathogenicity and/or those present in HGMD or literature. Literature searches were done in LitVar (https://www.ncbi.nlm.nih.gov/CBBresearch/Lu/Demo/LitVar/), Pubmed (https://pubmed.ncbi.nlm.nih.gov/), and Google Scholar (https://scholar.google.com/). Mutalyzer (https://mutalyzer.nl/) was used to check the syntax and names or abbreviations of the variants according to updated Human Genome Variation Society (HGVS) nomenclature.

### CTX allelic prevalence estimation

Carrier allele frequencies (AFs) of the final list of variants were used to calculate CTX prevalence across six gnomAD populations (AFR = African/African American, AMR = Latino/Admixed American, EUR = Non-Finnish European, FIN = Finnish, SAS = South Asian, EAS = East Asian) using the Hardy–Weinberg principle (p^2^ + 2pq + q^2^) [20, 35] under the assumption of mutual independence of the rare variants and full penetrance. Prevalence was calculated as the squared sum of the pathogenic alleles (q), and q was calculated from the carrier frequency (2pq) with p approximated to 1 [20]. The 95% confidence interval (CI) was calculated with the binomial formula using the Wilson method for each population’s list of risk alleles, as previously adopted [24, 36, 37].

### CTX variants search activity and geographic distribution via VarSome Insights

We used the VarSome Insights (VSI) service to gather genomic queries relevant to *CYP27A1* gene variation and generate an estimated CTX disease map based on the raw number of unique queries from professionals designated as healthcare providers (HCPs). Original profession labels were reclassified and grouped into fewer labels with the goal of distinguishing clinical/medical queries from research/other type of queries and to resolve word mismatches due to language or spelling differences. We further removed from the analysis HCPs queries that, based on the self-reported profession/position/title, were deemed more likely to be of research or nonclinical nature (eg, Research Scientist). Queries were gathered from January 1, 2021, to November 11, 2021.

## Results

We identified a total of 864 *CYP27A1* gene variants, of which the majority (40%) were missense. Forty-four variants were designated ClinVar pathogenic in gnomAD (12 frameshift, 13 missense, 8 canonical splice site, and 11 stop_gained), and 34 variants were considered pathogenic based on sequence consequence (17 frameshift, 3 start_lost, 10 stop_gained, and 4 canonical splice) (see Table 1).

We manually verified that the 44 ClinVar variants were assigned pathogenic in HGMD and ClinVar and that all 78 (44 + 34) were classified pathogenic by the VarSome algorithm (see Additional file 1). Altogether, pathogenic variants represented 9% of all *CYP27A1* variants in gnomAD. We then applied our REVEL bioinformatic workflow and identified 23 missense variants passing the disease-specific cutoff, thus predicted to be pathogenic. None of these 23 variants were predicted to be (likely) benign by the VarSome algorithm (see Additional file 1), and the vast majority were absent from the HGMD/literature. Of the 23 REVEL-predicted pathogenic variants, p.Met383Lys (VarID: 2-219678874-T-A, HGSV: c.1148T > A) was found with a higher AF (0.12%) in the AMR population compared with the remaining missense variants. With such AF and without reliable estimates for a parameter required to apply the Max Population AF filter [38], we opted to calculate two estimates for AMR prevalence: with and without the p.Met383Lys variant. Lastly, we manually searched for missense variants with a REVEL score below the cutoff but with supporting information for pathogenicity. We identified five additional missense variants, bringing the number of predicted pathogenic variants to 28 (see Table 1). The average REVEL score of these recovered variants was 0.78, which was within 0.5 standard deviations below the mean score of the HC pathogenic variants.

### CTX prevalence estimates

**Fig. 1:**
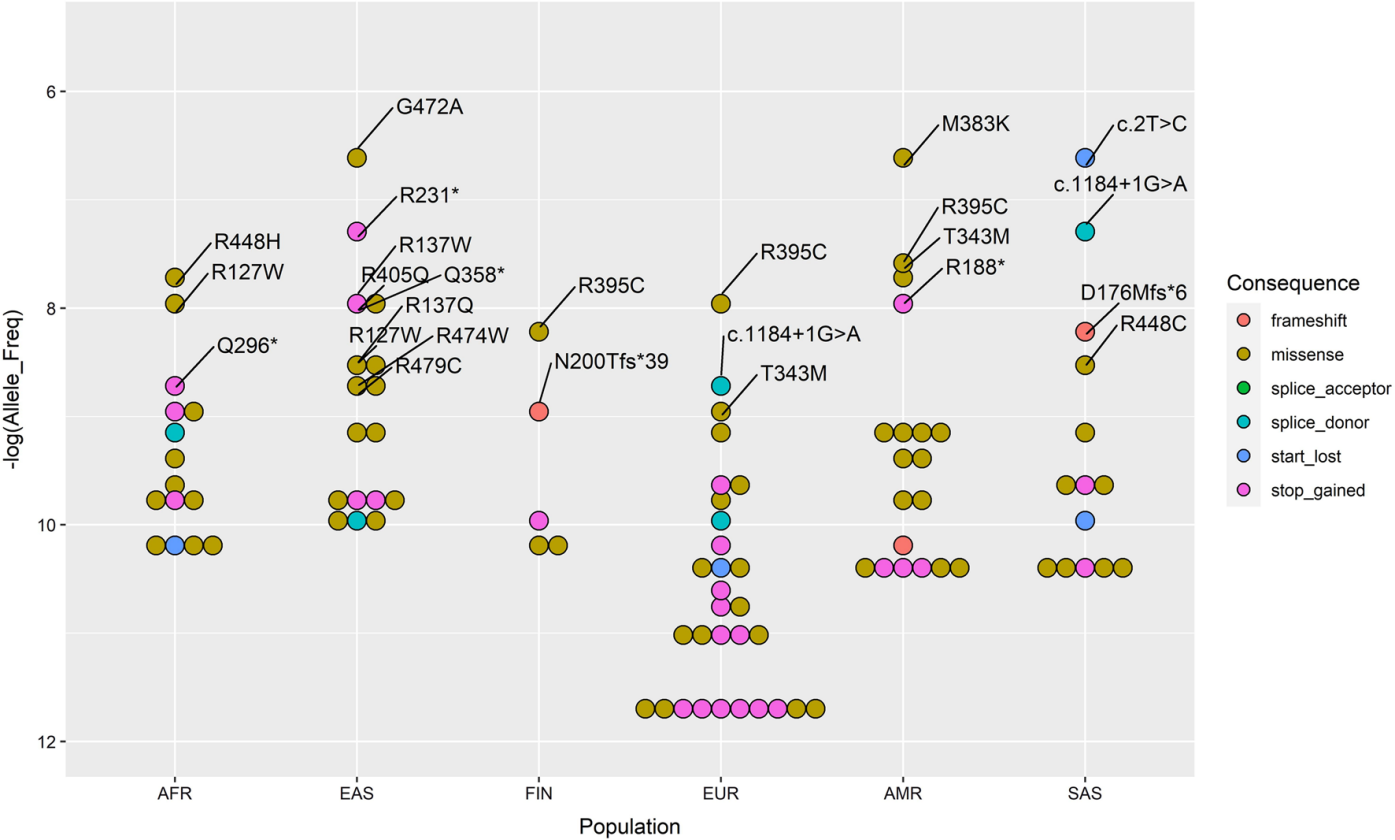
CTX alleles distribution from gnomAD. Distribution of the CTX alleles with labels for the top alleles (Allele_Freq > 1e-04) and color-coded by sequence consequence. Variant names follow the HGVS transcript or protein consequence. Amino acids are abbreviated by their one letter code

The final list of variants for the CTX prevalence calculation included 44 designated ClinVar pathogenic variants, 34 considered pathogenic based on sequence consequence, and 28 (including p.Met383Lys) predicted pathogenic variants by the in silico REVEL analysis (see Table 1 and Additional file 1). Using the gnomAD carrier AF of these 106 variants, we calculated CTX prevalence estimates, carrier frequency, and pooled AF by population (Table 2). We found the highest estimates in the EAS and SAS populations, ranging from 2.25 to 1.07 per 100,000 or from 1 per 44,407 to 1 per 93,084, with a pooled AF ranging from 0.00475 (95% CI 0.00187–0.01457) to 0.00328 (95% CI 0.00172–0.00764), respectively. The lowest prevalence estimate was found in the FIN population with 0.03 per 100,000 or 1 per 3,388,767 and a pooled AF 0.00054 (95% CI 0.00019–0.00169). These estimates were not substantially different from those calculated with only the 78 pathogenic variants excluding the variants only predicted pathogenic by the REVEL analysis (see Table 2). Intermediate prevalence estimates were found in the AMR, AFR, and EUR populations. In AMR without the variant p.Met383Lys (M383K), these estimates were 0.63 per 100,000 or 1 per 157,878 with a pooled AF 0.00252 (95% CI 0.00113–0.00653), while those with the M383K variant were 1.41 per 100,000 or 1 per 70,795, with a pooled AF 0.00376 (95% CI 0.00206–0.0082). In AFR, these estimates were 0.6 per 100,000 or 1 per 166,440, with a pooled AF 0.00245 (95% CI 0.00071–0.00898). In EUR, these estimates were 0.43 per 100,000 or 1 per 233,597, with a pooled AF 0.00207 (95% CI 0.0008–0.00671). Of note, estimates calculated without the predicted pathogenic missense variants showed very similar prevalence across AFR, AMR, and EUR, ranging from 0.25 to 0.21 per 100,000 or 1 per 393,497 to 482,603 (see Table 2). Figure 1 shows the most frequent alleles in each population. The missense variant G472A (VarID: 2-219679419-G-C, HGVS: c.1415G > C, p.Gly472Ala) and the nonsense variant (VarID: 2-219646907-T-C, HGVS: c.2T > C, p.Met1?) were the most frequent in the EAS and SAS populations. G472A was also recently identified in a dried blood spot sample together with the variant R405Q (VarID: 2-219679132-G-A, HGVS: c.1214G > A, p.Arg405Gln), which we also found in the top EAS alleles [19]. The missense variant R395C (VarID: 2-219678909-C-T, HGVS: c.1183C > T, p.Arg395Cys) remained the most frequent in the FIN and EUR populations, while in the AMR population was second to the missense M383K (VarID: 2-219678874-T-A, HGSV: c.1148T > A, p.Met383Lys). The missense variant R448H (VarID: 2-219679347-G-A, HGVS: c.1343G > A, p.Arg448His) was the most frequent in the AFR population.

**Table 2:** CTX prevalence estimates across six gnomAD populations

Model	Estimate	AFR	EAS	FIN	EUR	AMR*	SAS
ClinV-P + SeqCon-P	per 100,000	0.21	1.41	0.03	0.25	0.21	0.95
	1 per	472,468	71,089	3,946,059	393,497	482,603	105,299
ClinV-P + SeqCon-P + Revel-P	per 100,000	0.6	2.25	0.03	0.43	1.41/0.63	1.07
	1 per	166,440	44,407	3,388,767	233,597	70,795/157,878	93,084
	Carrier Frequency	0.0049	0.0095	0.00108	0.00414	0.00752/0.00504	0.00656
	Pooled AF (95% CI)	0.00245 (0.00071–0.00898)	0.00475 (0.00187–0.01457)	0.00054 (0.00019–0.00169)	0.00207 (0.0008–0.00671)	0.00376 (0.00206–0.0082)/0.00252 (0.00113–0.00653)	0.00328 (0.00172–0.00764)

*In the model ClinV-P + SeqCon-P + Revel-P, the two prevalence estimates correspond to with/without the M383K variant

AF, allele frequency; AFR, African/African American; AMR, Latino Admixed American; EAS, East Asian; EUR, European non-Finnish; FIN, European Finnish; SAS, South Asian

### CTX search activity geographic distribution

We leveraged VSI to infer a possible CTX disease geographic distribution estimate based on genomic variant queries through the VarSome search engine. This analysis was performed under the assumption that queries were potentially linked to clinical activity ultimately aiding the diagnosis or care of prospective CTX patients. In other words, it was assumed that HCPs from the selected clinical/medical specialties using VarSome in this manner were gathering *CYP27A1* variant pathogenicity information because a patient in their care was likely affected with CTX. Overall, queries were performed largely by clinical and research professionals using the platform to gather information on the *CYP27A1* gene and related variants. In 2021, we identified a total of 1243 queries, of which, 827 were from designated HCPs, representing 67% of all queries. After removing those under research or nonclinical professions, we considered 576 queries as a proxy for CTX clinical activity, of which half (n = 288) were from clinical geneticists and genetic counselors.

**Fig. 2:**
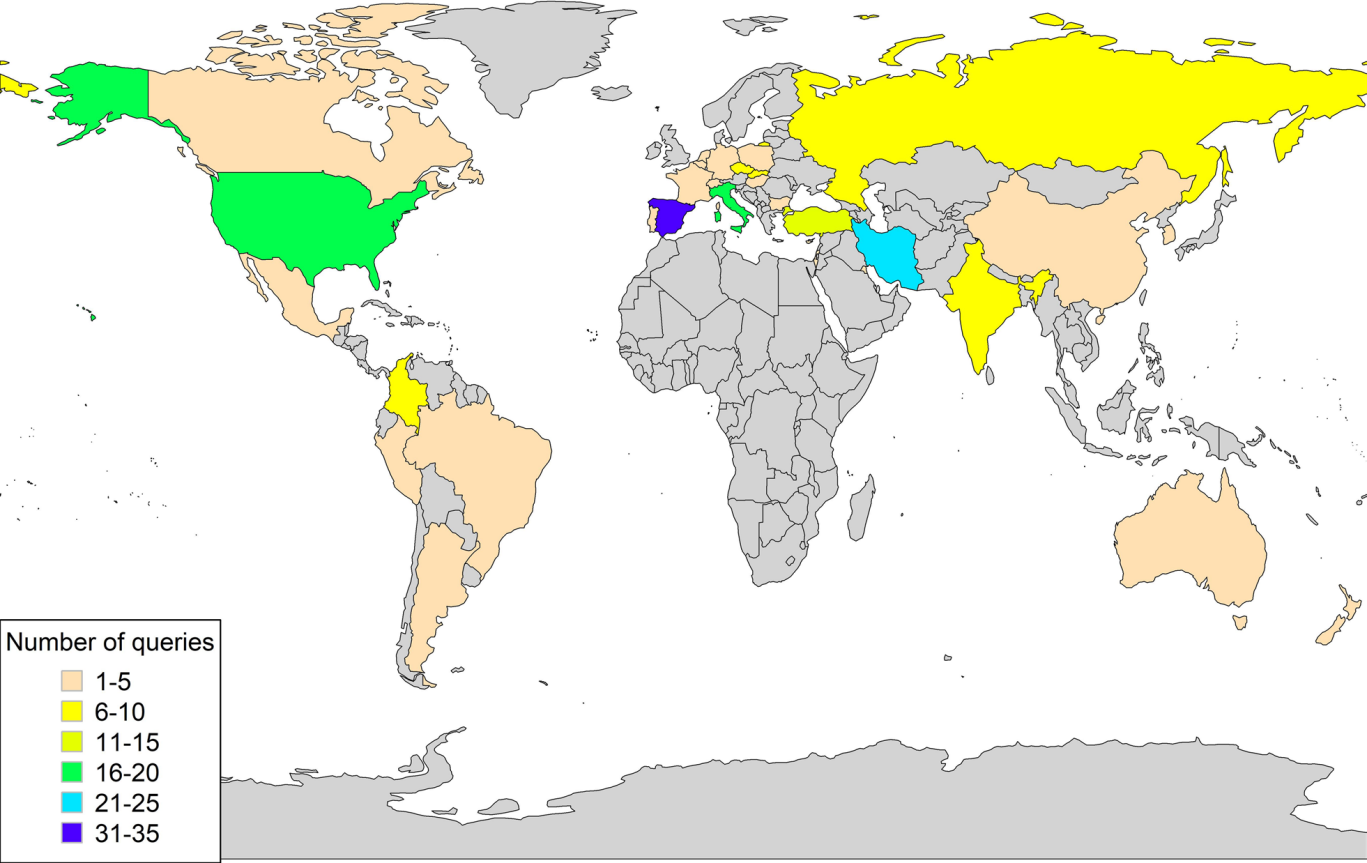
*CYP27A1* pathogenic variants world map. Geographic distribution of *CYP27A1* gene variants queries performed by HCPs of selected clinical/medical professions using the VarSome search engine. The map represents a proxy for clinical activity by country potentially linked to the diagnosis and care of prospective CTX patients. Grey color indicates no queries reported

Analysis of the final queries from HCPs showed that pathogenic, uncertain significance, and benign variants were equally represented (~ 33% each). The most queried variant was by far the missense P384L (NM_000784.4:c.1151C > T), which is consistent with its high population AF and several benign submissions in ClinVar. Among the top pathogenic variants, we found a similar number of queries for c.1184 + 1G > A, R127W (NM_000784.4:c.379C > T) and R395C/S (NM_000784.4:c.1183C > T/A), which are also found among the top alleles across gnomAD populations in our prevalence model (Fig. 1). Lastly, we mapped all the VarSome pathogenic HCPs queries (N = 187) to infer a possible geographic distribution of CTX disease and identified Spain as the top country with the highest number of *CYP27A1* variant queries, followed by Iran, Italy, USA, and Turkey (Fig. 2).

## Discussion

There is scientific consensus that CTX is clinically not well recognized, frequently has delayed diagnosis [17], and has a prevalence likely underestimated in the general population [20, 39], with less than 600 cases reported worldwide [12, 28]. In order to facilitate early diagnosis of this disease, we sought to calculate the most updated conservative and accurate estimates for CTX prevalence. We followed the approach of previous studies for estimating how common a monogenic autosomal recessive disease may be [20, 25, 33, 40, 41], and we leveraged the availability of a large genetic dataset, gnomAD. We developed a highly curated list of alleles in *CYP27A1* that have been reported to be pathogenic and used their bioinformatic characteristics to identify additional missense alleles of *CYP27A1* that are predicted to be pathogenic for CTX. We then applied the Hardy–Weinberg principle to estimate CTX risk in global populations. These analyses show that CTX is present in global populations at rates more common than previously appreciated. For the first time, we also attempted to geographically map CTX clinical activity worldwide, using a new emerging tool for genomic variation data sharing.

The belief that CTX is an exceedingly rare occurrence is not an uncommon scenario in the field of rare diseases. As for CTX, this is typically due to the intrinsic nature of the disease, which can show pleomorphism in clinical and laboratory features overlapping with other diseases, having a variable clinical course and symptoms onset, and lacking any clear genotype–phenotype correlations [3, 42]. The task of establishing an accurate estimate for how common CTX may be in the population becomes even more challenging considering the presence of a subpopulation of patients with a milder phenotype [27]. Interestingly, such individuals may carry the same variants (eg, p.Arg395Cys) that are found in patients with neurological involvement, suggesting that perhaps additional damaging mechanisms or genetic modifiers other than *CYP27A1* loss of function may be at play. Reinforcing this enigmatic picture is the report of a pair of siblings carrying the same pathogenic variants but being at opposite ends of the clinical spectrum, where one sibling developed rare spinal xanthomatosis and the other developed a mild form with minor tendon xanthomas [43]. In contrast, examples of common clinical manifestation are well documented. Bilateral cataracts are found in about 80% of CTX patients [16, 44] and interim analysis of patients recruited on the sole basis for having juvenile-onset idiopathic bilateral cataract shows a molecularly confirmed diagnosis in 1.5%-1.8% of the patients, representing a 500-fold increase in CTX prevalence in this subset of patients [45, 46].

Our findings largely support, expand upon, and refine previous genetic estimates based on the ExAC data and more limited variant inclusion [20]. We identified three levels for CTX prevalence. The highest estimates were found in the Asian populations at 1 per 44,407–93,084, which are in line with previous investigations (1:36,072–75,601) [20]. In both studies, the top variant remained the missense c.1415G > C (p.Gly472Ala), but with a higher carrier AF in gnomAD compared with the ExAC (0.0014 vs 0.0010) [20]. To our knowledge, the p.Gly472Ala variant has been reported in two patients: one of Asian origin carrying a homozygous mutation [5] and one in a newborn screening [19]. In the latter study, one sample was found positive for biochemical CTX biomarkers and compound heterozygous for two pathogenic variants in *CYP27A1* (c.1214G > A—p.Arg405Gln; c.1415G > C—p.Gly472Ala). While we do not have confirmation that this sample was from an individual of EAS ancestry, we speculate that this might be the case as p.Gly472Ala is unique to EAS and both alleles show high AF in EAS compared with the other ancestries in gnomAD. In such a scenario, the reported incidence of 1 per 32,000 from Hong et al. [19], might provide an independent and orthogonal validation of our findings in the EAS population.

An intermediate level of CTX prevalence was found in the AMR, AFR, and EUR populations. In AMR, although we obtained very close estimates (1 per 70,795–157,878) compared with the ExAC study (1:71,677–148,914) [20], differences in the number, type, and AF of variants may be found. For instance, we identified twice as many alleles, with the top variant p.Met383Lys showing a much higher AF (0.00124 vs 0.00078). We provided two prevalence estimates for AMR since it would be difficult to confidently include/exclude this variant from our analysis. Following ACMG/AMP guidelines, we could attempt to apply the BS1 criteria that is used to classify a variant as “likely benign” when its AF is greater than expected for the disorder [30, 47, 48]. However, reliable estimates for population prevalence and allelic heterogeneity would be needed to use the maximum credible population AF as filter [38]. A plausible strategy to leverage this filter would be to create a distribution of prevalence values and use two estimates for allelic heterogeneity, for instance, 10% (conservative) and 30% based on the most frequent allele found in CTX patients (c.1183C > T, p.Arg395Cys) [27]. By doing this exercise, we found that at 10% allelic heterogeneity, p.Met383Lys was retained only with a disease prevalence of 1:10,000–20,000 (penetrance 100%-50%), which is currently not supported by any clinical, epidemiological, or genetic study in the AMR population. At 30% allelic heterogeneity, we found that p.Met383Lys was retained with a disease prevalence of 1:100,000–180,000 (penetrance 100%-50%), which may be currently supported by genetic estimates from the previous CTX study (~ 1:70,000–150,000) [20]. However, it could be argued that if p.Met383Lys was as frequent as p.Arg395Cys, we would have expected at least a few CTX patients described in the literature carrying this allele.

Consistent with the strong literature evidence, we found p.Arg395Cys to be most prevalent in the EUR, FIN, and AMR populations. Also, this variant showed an increase in AF compared with the ExAC frequency (0.00051 vs 0.00017) [20]. Our estimates instead show a significant increase in prevalence for the AFR and EUR populations, that moved from 1:468,624 to 1:166,440 and from 1:461,358 to 1:233,597, respectively [20]. Again, the same top drivers were found between the two studies but with an increase in AF (p.Arg448His: 0.00041 vs 0.00031 in AFR; p.Arg395Cys: 0.00038 vs 0.00021 in EUR). As discussed above, such differences are not surprising, especially when considering the sizable difference in the number of individuals investigated between the two databases. In addition, we need to take into account improvements in the performance of in silico predictors and the evolving nature of clinical and functional information, which are both critical to variant classification.

Similar to the AF of p.Met383Lys, we have retained in our model the start_lost variant c.2T > C (NM_000784.4:p.?), which was found to be unique to the SAS population. While this type of variant may be considered always pathogenic, as it is expected to produce no protein, the clinical impact in practice can be heterogeneous, and alternative mechanisms for protein translation should be considered. In fact, it is notable to find this variant at the heterozygous state in four siblings of a South African family with a mild (no neurological involvement) CTX phenotype (see family #8) [27]. It is possible that future functional studies will be able to clarify the role of the c.2T > C allele in the pathogenesis of CTX or perhaps to rule out its involvement with direct implications for our SAS estimate. The lowest level of prevalence was found in the FIN population, which is in line with findings from the previous CTX study [20].

Lastly, with the purpose of increasing awareness, having a proactive approach to patient identification, and aiding early treatment intervention, we leveraged the VarSome platform to create a clinically relevant geographic map based on recent query activity. We found that most of the variant queries were from clinical geneticists/genetic counselors and that the most searched variants were consistent with our findings from the analysis of the gnomAD AF. We recognize that the proposed geographic map represents only a proxy to a possible clinical distribution of prospective CTX patients and that the difference in counts per country may also depend on or be limited to factors such as access and popularity of the VarSome search engine, country size, accessibility, and costs to genetic testing, as well as socioeconomic or political efforts to boost scientific progress in the fight against rare diseases. Nonetheless, our findings line up with patient reports from around the world (USA, Israel, Italy, Japan, the Netherlands, Belgium, Brazil, Canada, France, Iran, Norway, Tunisia, Spain, China, and Sweden; see CTX at https://www.ncbi.nlm.nih.gov/books/NBK1409/ and https://rarediseases.org/).

There are limitations and methodological assumptions underlying the approach in this study. We and others have applied the Hardy–Weinberg principle in our genetic risk calculations that assume that heterozygous individuals are not subject to selection, populations are at equilibrium with respect to allele and genotype frequencies, and random mating is observed. Also, the concept of AF and how rare an allele may be is strictly related to the size of the population under investigation; thus, with the availability of larger population-based datasets, estimates will change and become more accurate. Lastly, we recognize that although the adoption of filtering criteria and in silico tools to predict pathogenic missense with unknown or uncertain clinical significance is helpful and commonly used, there is currently no gold standard and these strategies could lead to the over- or underestimation of disease frequency. We attempted to mitigate overestimation by cross-referencing the pathogenic labels across different databases and sources and by applying strict filtering criteria with the goal of providing rather conservative estimates. We mitigated underestimation by manually curating missense variants that were filtered out by our bioinformatic workflow. Additional genetic variation that has not been accounted for in our calculation may be conferred by inframe deletions or insertions, intronic, noncanonical splice sites, and structural variants. Lasty, our approach assumed that all variants in the final model contribute to the risk of disease with 100% penetrance. While we are not aware of reports on *CYP27A1* pathogenic variants of reduced penetrance, we cannot exclude their presence.

In conclusion, our study, that includes additional variants, new informatics tools, and newer, expanded databases, supports and refines previous estimates for CTX disease risk at the population level and provides a novel prospective geographical map of CTX clinical activity worldwide. We confirm with this larger, more comprehensive study that CTX is more common than current worldwide patient estimates, and we highlight the most common pathogenic variants. We underscore the value of leveraging large and diversified population-based genetic databases to assess risk for inherited diseases. Such efforts, cross-referenced with other large-scale programs, such as newborn screening [19] and retrospective administrative claims studies [49], may provide the most accurate strategy to assess disease presence in a population. In turn, this will translate into greater awareness, better recognition, and early treatment intervention, which will directly benefit patients and caretakers.

## Supplementary Information

**Additional file 1.** Supplementary variants information.

## Data Availability

All data generated or analyzed during this study are included in this published article or uploaded as supplementary information and/or available in public open access databases described in the methods section.

## Abbreviations

CTX: Cerebrotendinous xanthomatosis
CDCA: Chenodeoxycholic acid
gnomAD: Genome Aggregation Database
ExAC: Exome Aggregation Consortium
HGMD: Human Gene Mutation Database
OMIM: Online Mendelian Inheritance in Man
HC: High-confidence
DM: Disease-causing mutation
ACMG: American College of Medical Genetics and Genomics
AMP: Association for Molecular Pathology
LP: Likely pathogenic
VEP: Ensembl Variant Effect Predictor
SVI: Sequence Variant Interpretation
HGVS: Human Genome Variation Society
AF: Allele Frequency
AFR: African/African American
AMR: Latino/Admixed American
EUR: Non-Finnish European
FIN: Finnish
SAS: South Asian
EAS: East Asian
CI: Confidence Interval
VSI: VarSome Insights
HCP: HealthCare Provider
